# Second and third harmonic generation in topological insulator-based van der Waals metamaterials

**DOI:** 10.1038/s41377-025-01847-5

**Published:** 2025-09-22

**Authors:** Alessandra Di Gaspare, Sara Ghayeb Zamharir, Craig Knox, Ahmet Yagmur, Satoshi Sasaki, Mohammed Salih, Lianhe Li, Edmund H. Linfield, Joshua Freeman, Miriam S. Vitiello

**Affiliations:** 1 NEST, CNR-NANO and Scuola Normale Superiore, 56127 Pisa, Italy; 2 School of Electronic and Electrical Engineering, University of Leeds, Leeds, LS2 9JT UK; 3 School of Physics and Astronomy, University of Leeds, Leeds, LS2 9JT UK

**Keywords:** Metamaterials, Other photonics

## Abstract

High-order harmonic generation (HHG) in solids—the frequency up-conversion of an optical signal—is governed by symmetries. At terahertz (THz) frequencies, HHG is a key technology to access high-frequency spectral windows that are usually difficult to cover using conventional solid-state laser technologies. This effect has been recently exploited in graphene, where HHG has been demonstrated, albeit only at odd multiples of the driving frequency owing to its inherent centro-symmetry. In topological insulators (TIs), the combination of spin–orbit interaction and time-reversal symmetry create an insulating bulk state with an inverted band order, inseparably connected with conducting surface states. TIs have been predicted to support unconventional high harmonic generation from the bulk and topological surface, which are usually difficult to distinguish. However, no experimental results have been reported, so far. Here, we exploit the strong optical field amplification provided by an array of single or double split ring resonators, with embedded Bi_2_Se_3_ or (In_*x*_Bi_(1−*x*)_)_2_Se_3_/Bi_2_Se_3_ Van der Waals heterostructures, to achieve up-conversion in the 6.4 (even)–9.7 (odd) THz frequency range. This results from bulk centro-symmetry (odd states) and symmetry breaking in the topological surface states (odd and even).

## Introduction

Dirac materials (DM) possess gapless, linear energy band structures, and have massless Dirac carriers that dominate their optical and electronic properties at the few atomic monolayers’ level^1–3^. They also display extraordinary nonlinear-optical properties^4^, and graphene is, in this respect, the most peculiar example, owing to its Dirac carriers’ electrodynamics^5^. When light is absorbed in graphene, electron-electron interactions induce electron heating on a 10–100 fs timescale, which is followed by electron cooling, typically involving the emission of phonons on a picosecond timescale^6,7^. Over the past decade, the non-linear optical properties of graphene have been extensively investigated, and a wide range of associated mechanisms were reported, such as saturable absorption^8,9^, self-phase modulation^10^, and four wave-mixing^11^. The ultrafast intraband dynamics^6,7^, the centrosymmetric crystal nature of the honeycomb single-layer graphene structure^12^, and the inherent giant optical third-order nonlinearities^13,14^ have also led to the demonstration of frequency up-conversion^13–18^— high harmonic generation (HHG)—in a broad spectral range from the ultraviolet^15^ to the mid-IR^16^, and down to the technologically-relevant terahertz (THz) frequency range, even at room temperature and in large-area synthetic films. This is a consequence of the third-order non-linearity (*χ*^(3)^ ~ 10^−9^ m^2^ V^−2^), which is almost seven orders of magnitude larger than that of typical semiconductor heterostructure lasers (7 × 10^−16^ m^2^ V^−2^)^2,19,20^, and significantly larger than that of alternative materials exploited in THz photonics and electronics^21^. In the THz range, HHG with conversion efficiencies ~1000 higher than the pristine film, were reported in silicon films embedded in metasurfaces^22,23^, exploiting n-doped silicon in the so-called perturbative regime or in impact-ionization driven processes. At THz frequencies, in DMs, the nonlinear effects responsible for the observation of HHG can usually be ascribed to the collective thermodynamic response of the free carriers to the driving field^24^. Crucially, unlike in other frequency domains, HHG can be induced at moderate fields (~≤10 kV/cm) achieving conversion efficiencies in the 1–10% range^25^, when using pump sources in the low THz frequency, and hence HHG does not require dedicated research facilities, such as the use of bulky laser systems^24^ or accelerator-based sources^26^. Conversely, in the high ≥2 THz frequency range, to overcome the small Drude weight of DM^27,28^—a parameter that quantifies the intraband absorption strength of the semi-metallic Dirac free carriers, and is limited by their number, ~10^−4^ times lower than in noble metals^29^—either plasmonic structures or micro-resonators must be used to confine the THz fields to deeply subwavelength volumes^30^, and to reach electric field amplification factors up to two order of magnitude larger than in a bare film. The combination of plasmonic confinement and resonant field enhancement in a DM^18^ recently led to third HG in graphene using an high power (>2 W) THz quantum cascade laser (QCL) as a pump^31^. The inherently atomic-layer thickness of bi-dimensional DMs makes them an ideal material platform for developing HHG-based sources, since it prevents the occurrence of decoherence/dephasing, due to asynchronous harmonic build-up in the nonlinear medium, ultimately leading to weaker and unclean frequency up-converted lines of defined parity^32^.

Topological insulators (TIs)^2,3^ have attracted considerable interest owing to their peculiar characteristic of having an insulating bulk state and highly conductive, topologically protected surface states with a Dirac-like dispersion^33^. Dirac carriers, in the topological surface states, exhibit dissipation-free transport, owing to spin-momentum locking and suppressed back-scattering^34^. Furthermore, they have been proven to sustain robust long-lifetime collective surface excitations, i.e. plasmon-polaritons, at both THz^35–37^ and mid-IR^38,39^ frequencies, thus prospecting new applications as the development of thermoelectric cryostats^40^, magnetic storage devices^41^, spintronic emitters^42^ and topological quantum computing elements^43^. Second- and third harmonic generation (SHG and THG) have been recently reported in Bi_2_Se_3_, a widely studied TI^44,45^. Unlike graphene, SHG in TIs is not forbidden, as Bi_2_Se_3_ has a non-zero second order susceptibility, stemming from inversion symmetry-breaking in the surfaces states^46^ and phonon effects. Essentially, in Bi_2_Se_3_, with a centrosymmetric bulk, the second-order response of THz-SHG can arise only when the optical response is dominated by the topological surface states^44,47,48^. However, in a recent report^45^, demonstrating THG in the THz range in a Bi_2_Se_3_ film, SHG was not observed. The absence of SHG usually results from a significant interplay between surface and bulk states, e.g. when the Coulomb interaction with the bulk carriers becomes the most proficient path for the ultrafast heating-cooling dynamics, mechanisms that govern the nonlinear response^45^. In an alternative picture^44^, the suppression of second-order processes was ascribed to the uneven population in the topological surface states, in twinned crystal domains^44^. This highlights the importance of developing sophisticated and systematic techniques to synthesize large-area and scalable TI films, with accurate control of the orientation and the quality of the crystal growth, particularly when surface symmetry breaking is sought. Recently, molecular beam epitaxy (MBE) has emerged as a powerful method for growing Bi_2_Se_3_ films of controlled thickness and composition^35,49–51^, and for devising tunable topological surface states through atomically controlled multilayering of different TI compunds^50,52^. As an example, the deposition of an insulator ultrathin film adjacent to the TI surface^50^ was revealed to be a promising strategy for achieving a robust insulating bulk, with surface states possessing a tunable Dirac point. Indeed, the incorporation of a small Sb amount in Bi_2_Te_3_ allowed for the tuning of the energy of the Dirac point^52^ in the topological surface state (TSS) of the Bi_2_Te_3_^52^.

In this work, we exploit the strong optical field amplification provided by a metamaterial array of single or double split ring resonators, embedding Bi_2_Se_3_ or a set of quantum-designed (In_x_Bi_(1-*x*)_)_2_Se_3_/Bi_2_-based van der Waals (vdW) heterostructures having engineered thicknesses, both grown by molecular beam epitaxy, to innovatively tailor, either odd (governed by bulk centro-symmetry) or even and odd orders frequency up-conversion in the THz frequency range, induced by symmetry breaking in the TSSs. This is achieved by optically pumping the resonator array with a high-power THz QCL. The mode confinement and field enhancement in the split gap/gaps, combined with the high-power density of the selected QCL (~≤1 kW/cm^2^), is essential to activate THG and SHG in the largely unexplored high THz (>3 THz) frequency range. The thin (In_*x*_Bi_(1−*x*)_)_2_Se_3_ virtual substrate mitigates the formation of defects at the substrate–TI interface, which can lead to the formation of trivial interface states. This increases the proportion of carriers in the TSS, and improves its overall quality, leading to SHG, a phenomenon not allowed in the Bi_2_Se_3_ sample, since the interaction of Dirac and bulk carriers prevents the symmetry breaking needed to allow the second-order optical process, even if the TI surfaces remain protected^45^.

## Results

### Bi_2_Se_3_ split ring resonator: design, nanofabrication and characterization

We initially engineer an array of micrometric circularly shaped single-split ring resonators (SSRR) on a THz transparent SiO_2_/Si substrate. The resonator geometry was optimized to match the imping QCL frequency (central bandwidth at 3.25 THz), while exploiting the field enhancement in the split gap of each individual SSRR^53,54^, following a design optimization procedure presented elsewhere^31,53^. In circular SSRRs, the two ends of the split gap capacitors set the dipolar length, and hence the fundamental mode wavelength. On SiO_2_, a split gap size of 1–1.5 µm is required to target the ~3.25 THz frequency of the pump laser adopted in the present work^31^. On the other hand, the field amplification itself is inversely proportional to the gap size but ultimately depends on the overall ring losses, that arises from the complex interplay of the geometrical parameters, such as ring thickness, radius, and periodicity^31^.

**Fig. 1:**
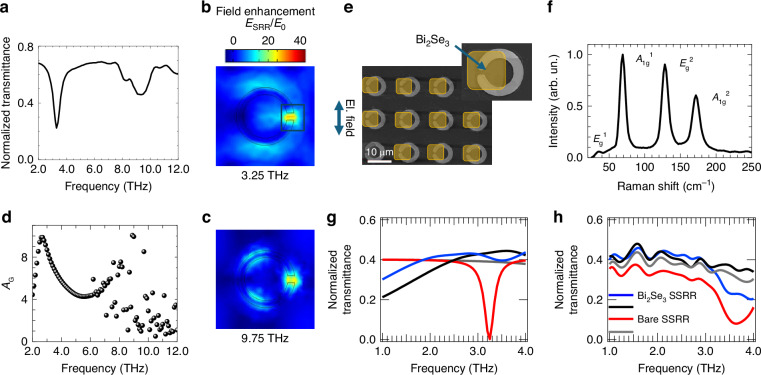
Optical response of the SSRR comprising Bi_2_Se_3_. **a** Simulated transmittance, calculated using a finite element method (FEM), of the Bi_2_Se_3_-SSRR, for an electric field polarization parallel to the dipolar split gap. **b** and **c** two-dimensional (2D) profiles of the electric field (*E*_SRR_) calculated using FEM simulations of the Bi_2_Se_3_-SSRR for the **b** fundamental and **c** third harmonic frequency modes, normalized by the field on the surface outside the resonator, E_0_ (zero enhancement region). **d** Amplification factor (*A*_G_) as a function of frequency, calculated for the Bi_2_Se_3_-SSRR, defined as the ratio of the average electric field on the split ring resonator plane to the average electric field impinging on an unpatterned SiO_2_/Si substrate adopting the same illumination boundary conditions and input settings in the simulator module. **e** Scanning electron microscope (SEM) image of the fabricated Bi_2_Se_3_ embedded SSRR array. The yellow areas (false color) highlight the patterned Bi_2_Se_3_. **f** Micro-Raman spectra measured on the Bi_2_Se_3_ SSRR, after transfer and processing. **g** Normalized transmittance of the Bi_2_Se_3_-integrated (blue, black curves) and bare (red, gray) SSRR measured using time-domain spectroscopy (TDS, Terascan by Menlo Systems), in a nitrogen-purged environment, with the dipole of the SSRR oriented parallel (blue, red) and perpendicular (black, gray) to the linearly polarized beam of the TDS transmitter. Each transmittance curve was obtained by Fourier transformation of the time-domain traces, normalized by the transmission through the SiO_2_/Si substrate. The dotted line marks the blue shift of the blue curve. **h** Calculated transmittance (COMSOL Multiphysics), with the same color code as panel (**g**)

The integration of the Bi_2_Se_3_ in the resonator array requires an accurate model that accounts for the complex optical constants of the TI, to evaluate the field enhancement and the optical response. Here, to mitigate the optical screening of the metallic SSRR array, and the consequent resonance bleaching associated with the semi-metallic character of the Dirac carriers in the TIs, we integrate the Bi_2_Se_3_ only in the SSRR split gap. The growth and characterization of the large area ~20 nm-thick Bi_2_Se_3_ adopted in the present work is described in ref. ^35^. The near field optical mapping of this film shows that the material sustains propagation of Dirac plasmon polaritons^35^, the dispersion of which is dominated by the Dirac carriers hosted in the surface topological linear bands, the 2D massive electrons in the trivial surface bands, and, the dielectric response of the thin bulk layer^35,36^. Hence, in agreement with the methods of refs. ^35–37^, its total conductivity is given by the sum of the bulk conductivity (*σ*_bulk_), the massive surface carrier conductivity (*σ*_2DEG_), arising from the Shockley-type band-bending at the surface^35^, and the conductivity of the Dirac-like states (*σ*_DM_). We then calculate the total transmittance (Fig. 1a) of the Bi_2_Se_3_/SSRR using finite element method (FEM) electromagnetic simulations (COMSOL Inc.), by assigning the total optical conductivity (see see Supplementary Information (SI), section S1), to a 5 × 5 µm^2^ rectangular area around the gap, considered as a transition boundary surface (see Fig. 1b). The Bi_2_Se_3_/SSRR calculated transmittance shows a resonant absorption dip (Fig. 1a) matching the frequency of the pump *ν*_o_ = 3.25 THz, and a quality factor *Q* = 5.1 ± 0.2 for the present design, with an additional broader dip located at 3*ν*_o_.

The 2D map of the electric field enhancement in the SSRR (amplification factor *A*_G_) at resonance, is reported in Fig. 1b. Here, *A*_G_ is defined as the ratio between the average electric field on the TI-embedded SSRR surface and the average electric field arising when plane wave illuminates an unpatterned surface (i.e. a SiO_2_/Si substrate), assuming the same incoming power and boundary conditions on the input port in the simulation. In the gap area, *A*_G_ is more than one order of magnitude larger than the absorption in an unpatterned film. This leads to the formation of an intense hotspot in the TI^55,56^, owing to the dipolar resonant mode sustained by the two arms of the split gap, that maximizes the light–matter interaction. This, in turn, shifts and enhances the nonlinear terms of the Bi_2_Se_3_ optical conductivity at the QCL pump frequency, increasing the HHG conversion efficiencies^31^. Interestingly, the SSRR has resonant modes even at harmonics higher than the fundamental, as shown in Fig. 1c for the third harmonic, boosting the overall THG efficiency^14^. The presence of a third-order resonance in the transmittance, having a quality factor *Q*_3_ = 5.3 ± 0.2, is reflected in the frequency dependence of the field enhancement (see Fig. 1d), which, in fact, shows a second frequency range of amplification, also around the third-harmonic frequency range.

We then fabricated the designed Bi_2_Se_3_/SSRR structure. We first realized the metallic SSRR array, of size 5 × 5 mm^2^, on a SiO_2_/Si substrate, using direct laser-writer photolithography, followed by deposition and lift-off of a Cr (5 nm)/Au (100 nm) metal bilayer. Next, the MBE Bi_2_Se_3_ film was transferred on top of the patterned sample, using a PMMA-assisted wet transfer method^57^. The transfer process allows for a reliable and conformal covering of the resonator gap area. Any potential bending effect of the Bi_2_Se_3_ film, across the metal edges of the ring is not affecting the SSRR optical response, since the presence of a suspended portion of the film does not change the electric field distribution in the resonator gap (see SI, Section 2). Finally, a second optical lithography step was performed to define and mask the gap region area, and then to remove the Bi_2_Se_3_ film from all areas surrounding the gap (Fig. 1e). To assess the quality of the Bi_2_Se_3_ film after the transfer and processing, we performed micro-Raman spectroscopy directly on the Bi_2_Se_3_ integrated into the resonator (Fig. 1f). Raman spectra (Horiba, Explora Plus microscope) were measured using a 532 nm laser delivering 1 mW optical power, focused to a ~ 2 µm spot. Three visible peaks associated with the three prominent vibrational modes of Bi_2_Se_3,_ namely A_1g_^1^, A_1g_^2^ and E_g_^2^, are retrieved at 69.0, 171.8, and 128.5 cm^−1^ wavenumbers, respectively, with a weaker peak at 37.1 cm^−1^ assigned to the E_g_^1^ mode; this is in agreement with previous characterizations of the as-grown MBE wafer^35^, confirming that the good crystalline quality of the MBE synthesized films was not affected by the processing.

The transmittance (Fig. 1g), measured using time-domain spectroscopy (TDS), shows a resonant response, visible only when the axis of the dipolar resonance of the ring is oriented parallel to the polarization direction of the probe beam, thus proving the resonator sensitivity to the incoming field polarization. When we compare the transmittance of the Bi_2_Se_3_-integrated SSRR array with that retrieved on the pristine resonator array having an identical design, we observe a weaker (~22%) and blue-shifted (0.20 THz) resonance, and a slight (~10%) decrease in the transmittance of the reference SSRR, compatible with the 13% Bi_2_Se_3_ coverage of the surface, that contributes to a change in the total reflectivity.

Both frequency shift and resonance broadening arise from the optical response of the Bi_2_Se_3_ film. Indeed, the semimetal character and the inductive response in the 2D material embedded SRR result in a “screening” effect of the metallic resonator, with the consequent resonance weakening due to the absorption losses in the Bi_2_Se_3_ material.

The frequency shift and resonance broadening of the optical response is clearly visible in Fig. 1h, which shows the simulated transmittances, that are in broad agreement with the experimental data (Fig. 1g). Notably, the field enhancement is only slightly affected by the presence of the TI film, in both its frequency behavior and magnitude (see SI, S2). This proves, as expected, that the resonator acts as a field enhancer, independently of the active film’s presence, indicating that the optical amplification of the resonator can be reliably assessed in both the pristine and the TI-integrated cases. However, in order to match the desired pump frequency and assess accurately the effective electric field amplitude on the TI film, the full complex conductivity must be included in the resonator design.

### Third harmonic generation (THG) in externally pumped Bi_2_Se_3_/SSRR array

**Fig. 2:**
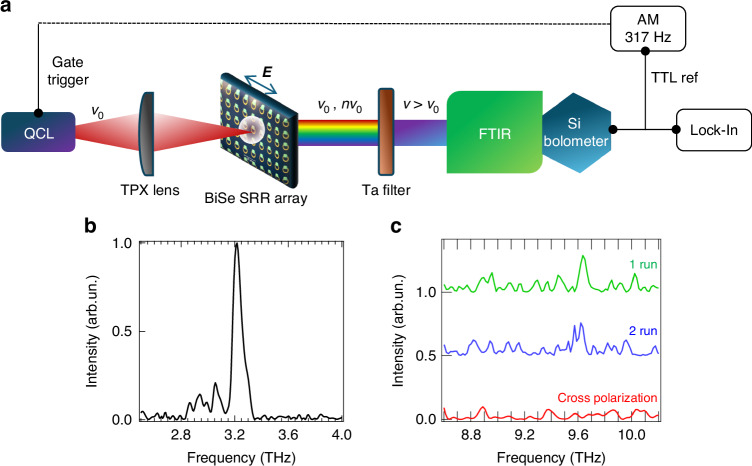
High harmonic generation in the optically pumped resonator array. **a** Schematic diagram of the experimental system for investigating SHG and THG. Light from a single-plasmon QCL, emitting 2.5 W optical power, into free space, is focused on the resonator array. The SHG and THG signals are isolated after filtering the THz QCL beam with a 6 THz high-bandpass Ta-filter. **b** Fourier transform infrared (FTIR) emission spectrum of the QCL pump source, acquired under vacuum, with a 1 cm^−1^ spectral resolution. **c** Emission spectrum at the third harmonic frequency, measured on the optically pumped Bi_2_Se_3_-SSRR from two different fabrication runs (green and blue), after filtering the QCL with the Ta-filter. Spectra were measured in step-scan mode with a spectral resolution ~1 cm^−1^ and aperture size of~5 mm, with the split-gap dipole of the SRRs in the array oriented parallel (green and blue) and perpendicular (red) to the laser linear polarization direction

To demonstrate THG in the Bi_2_Se_3_ embedded resonator, we performed the experiment sketched in Fig. 2a, in which we probed the emission from the Bi_2_Se_3_ SSRR array while pumping it with an intense (~1 kW/cm^2^) beam illumination. Signals emitted by the Bi_2_Se_3_-SSRR were acquired in step-scan mode, under vacuum and with a resolution of 1 cm^−1^, after filtering the frequency of the QCL, with a 2-mm-thick high-bandpass Ta crystal filter. We first oriented the dipolar mode axis of the SSRR along the polarization axis of the QCL, then perpendicularly. A peak at ~9.75 THz, signature of THG, at the third-order harmonic of the pump beam (Fig. 2c, green and blue curves, acquired on two different samples from separated fabrication runs) was only measured when the polarization of the pump beam coincides with the dipolar orientation of the SSRR, illustrated in Fig. 1b. No evidence of possible signals at the 3^rd^ (or 2^nd^)-harmonic frequency was retrieved when the resonator was pumped along the orthogonal direction (cross-polarization pumping). Although ≥7 h long scans were required to acquire data for both samples, a persistent THG signal was measured ~(1.3 ± 0.3)×10^−5^ less intense of the pump; this conversion efficiency value was determined after accounting for the acquisition conditions (including the gain conversion of the lock-in amplifier and FTIR aperture diameter) and it is comparable with the conversion efficiency reported in single layer graphene^31^. No signature of SHG was observed. This is in agreement with previous reports^45^, showing only odd-harmonic generation in Bi_2_Se_3_; indeed, it is theoretically predicted^44^ that SHG would require symmetry breaking in the TSS. This stems from a topological protection strong enough to guarantee the full isolation of the optical response of the surface states from those in the bulk^44^. Specifically, the TI surface state in the present sample, with a mobility measured on the as-grown film of 1500 ± 100 cm^2^/V s^35^, was shown to be strongly affected by the presence of lower mobility carriers in the trivial surface state, i.e. the massive 2DEG, and by the bulk conductivity—this was revealed by the absence of Landau levels quantization in low-T magneto-transport, up to an 8 T magnetic field^35^.

### Third harmonic generation: modeling of the optical response in the Bi_2_Se_3_ resonators

Since the investigated Bi_2_Se_3_ samples did not show any evidence of SHG, we restrict our modeling to third order processes. We calculate the conversion efficiency by implementing the same analytical model developed for graphene-SSRR arrays^31^. We assume that the non-linear optical response of the Bi_2_Se_3_ film stems exclusively from the hot-electron dynamics in the massless Dirac carriers in the TSSs^4,45^. The bulk affects the overall resonator response, as shown by the resonance shift/broadening and by the modulation of the field enhancement. However, it has also been recently reported^45^ that the bulk carriers provide an alternative ultrafast dissipation channel for the hot electron temperature, through surface-bulk Coulomb interactions. This property induces an increase in the up-converted signal amplitude of more than two orders of magnitude^45^ compared to the case of widely studied graphene samples^58^.

In the external pump scheme employed in the present work, the THG conversion efficiency (CE) is defined as the variation of the sample transmission normalized by the linear transmission, ${CE}={(T}_{3}-{T}_{0})$*/*$\,{T}_{0}$, and is evaluated through the Tinkham formula^59^, as follows:

1 $$\left[{T}_{0},{T}_{3}\left(\nu \right)\right]=\frac{4{n}_{{sub}}}{{Z}_{0}^{2}{\left|\frac{{n}_{{sub}}}{{Z}_{0}}+\left[{\sigma }_{0},{\sigma }_{3,{tot}}\left(\nu \right)\right]\right|}^{2}}$$

where Z_0_ is the vacuum impedance and *n*_*sub*_ is the refractive index of the substrate. Following Eq. 1, CE is then related to both the linear (*σ*_*0*_ = *σ*_*DC*_ + *σ*_*2DEG*_ + *σ*_*Bulk*_) (see SI) and the field-driven nonlinear (${\sigma }_{3{tot}}={\sigma }_{0}+{|{E}_{0}\left(\nu \right)|}^{2}{\rm{\times }}{\sigma }_{3}\left(\nu \right))$ optical conductivities. In the latter expression, ${\sigma }_{3}\left(\nu \right)$ is the non-linear Kerr optical conductivity of the Bi_2_Se_3_ topological carriers, calculated accounting for the hot-electron temperature (T_e_) dependence of the Drude coefficients^60,61^, and including the contribution of the plasmonic frequency of the resonator array by substituting the frequency ν with the effective, spectral frequency ${{\rm{\nu }}}_{{\rm{eff}}}=\left({{\rm{\nu }}}^{2}-{{\rm{\nu }}}_{0}^{2}\right)/{\rm{\nu }}$ (see SI, section S3-5). To mimic the operation of our external pump, comprising a QCL driven in pulsed mode at ~kHz repetition rate and with pulse duration ~µs, T_e_ is evaluated for steady state illumination^31,58,61^, i.e. when the optical beam is either continuous wave or with a pulse duration much longer than the cooling time (~ps). Under this assumption, T_e_ depends on the ratio between the incoming pump power, the cooling time of the TI film (τ_cool_ ~ 3.5ps^62^), and the heat capacitance of the hot fermions of the Bi_2_Se_3_ (C_Bi2Se3_ ~ 0.16 µWm^−2^sK^−1^). Remarkably, unlike the case of few-ps pulsed pumping^6,45,63^, in this condition, namely with ~10^7 ^ cycles of the impinging laser mode driving the hot carrier populations, a slower cooling channel is not detrimental for efficient THG, but rather helps to build up higher T_e_, with a consequent stronger modulation of the T_e_-dependent conductivity, which in turn leads to a larger harmonic generation efficiency. In extracting the electronic temperature, we neglect the power dissipation through the substrate, and we assume that the thermalization within the electronic system dominates over alternative energy relaxation channels, as for example the heat transfer to the substrate^45,58,64^.

**Fig. 3:**
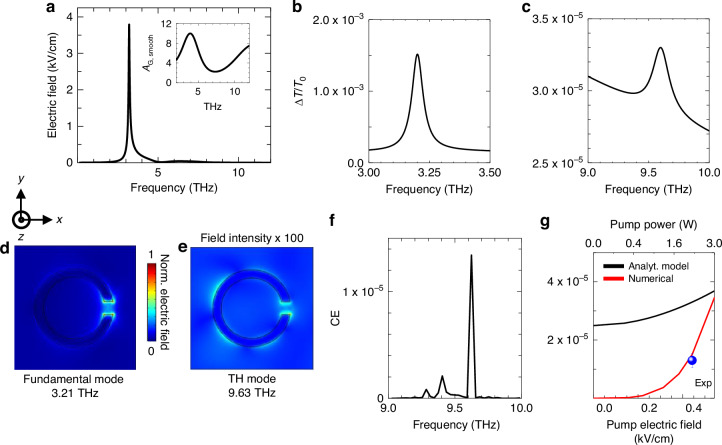
Analytical model of THG in Bi_2_Se_3_ SSRR. **a** Electric field incident on the Bi_2_Se_3_ SSRR as a function of frequency, calculated through the convolution of the field enhancement shown in the inset, and the optical beam intensity, modeled assuming a 2 W Gaussian-peak emission power focused on a 700 μm diameter circular spot. **b** and **c** ΔT/T_o_ as a function of frequency in the pumped Bi_2_Se_3_ SSRR, assuming the incident effective electric field from (**a**), and following the analytical model illustrated in the text, plotted **b** at the fundamental QCL frequency *ν*_0_, and **c** at 3*ν*_0_. **d** and **e** Two-dimensional (2D) profiles of the emitted electric field from the Bi_2_Se_3_ SSRR, calculated according to the refined numerical model illustrated in the main text at **d** the fundamental resonance frequency (3.21 THz), and **b** the third harmonic frequency, (9.63 THz). **f** Third harmonic conversion efficiency (CE) as a function of frequency, calculated using the surface current density numerical model, assuming an input power of 2 W. **g** THG efficiency, Δ*T*/*T* (3*ν*_0_), in the Bi_2_Se_3_ SSRR, as a function of the impinging electric field (bottom axis) and total power (top axis), calculated assuming an incident beam diameter of 700 μm, with simplified analytical (black) and refined numerical (red) models, respectively. The blue circle represents the CE value retrieved from the experiment

Figure 3a shows ${\left|{E}_{0}\left(\nu \right)\right|}^{2}$, that is the effective electric field driving the nonlinear response. It is given by the convolution, as a function of the frequency, of the field enhancement, numerically extracted from the FEM simulation data in Fig. 1d both at the fundamental and at the third harmonic frequency (and plotted in the inset of Fig. 3a using a 10-point, adjacent-averaging smoothing procedure), and the optical electric field of the external beam, modeled as a quasi-monochromatic radiation field delivering 2 W optical power, focused on a 0.4 mm diameter circular spot. The effective electric field in Fig. 3a was used to calculate the third harmonic CE as a function of frequency (Fig. 3b, c).

Owing to the photo-induced *T*_e_ dependance of the conductivity, the calculated transmission variation $\Delta T$*/T*_0_ at the fundamental frequency *ν* = *ν*_0_ increases (Fig. 3b), a phenomenon ascribed to the absorption saturation already reported in DM^8^, and specifically in TIs^65,66^. In addition, another peak-like increase is predicted in $\Delta T$*/T*_0_ at *ν* = 3*ν*_0_, corresponding to a CE ~ 3.3 × 10^−5^ for THG emission (Fig. 3c); this is the only high-order harmonic term included in our model, and is also boosted by the field enhancement in the CSSR. While the mode at *ν* = 3*ν*_0_ is always retrieved in the investigated SRRs, likely due to symmetry reasons, the same does not happen for the mode at *ν* = 2*ν*_0_. Noticeably, the frequency dependent $\Delta T$/*T*_0_ also shows a baseline of ~2 × 10^−5^ around the third harmonic peak. This arises from the non-zero values of the nonlinear optical conductivity, at all frequencies *ν* ≠ 3*ν*_0_, which lead to small, non-zero background contributions to $\Delta T$/*T*_0_.

To better account for the role of the field amplification of the SSRR to the THG efficiency, we implemented an alternative, more sophisticated, numerical model to predict the THG process in the plasmonic structure, by following the approach proposed in^67^. This method allows to extract the THG efficiency as an output parameter directly from simulations, by setting up the equations for the third harmonic generated field in the software module. The simulation layout comprises the same unit cell for the resonator, as used in the linear simulation of Fig. 1. The method is then based on the assumption that the Bi_2_Se_3_ TI film is a nonlinear surface current generator, defined as $J={\sigma }_{0}{E}_{{{\rm {TH}}}}+{\sigma }_{3}{E}_{{{\rm {FH}}}}$, where *E*_FH_ and *E*_TH_ are the electric fields induced at the fundamental frequency and at the third harmonic frequency, respectively, with *σ*_0_ being the total Bi_2_Se_3_ conductivity, and *σ*_3_ the third-order non-linear conductivity, again expressed as the *T*_e_-dependent Kerr nonlinear term^68^ (see SI).

Within this method, the electromagnetic simulator can compute the CE of THG, by solving the nonlinear Maxwell equations at the third harmonic frequency. By exciting the resonator structure externally (from the top), it can be verified that it possesses the desired resonant mode (Fig. 3d). This result is implemented by using an electromagnetic input port, for which the power density—the most critical parameter to estimate the conversion efficiency—is a freely defined parameter. To extract quantitatively the CE, defined as the ratio between the delivered power at the third harmonic and the input power, the input port power is set to match the power density range used to illuminate the Bi_2_Se_3_-SSRR. The electric field profile at the third harmonic frequency (Fig. 3e) shows a distribution similar to that of the fundamental mode, although is characterized by a field intensity more than two orders of magnitude lower. The calculated frequency dependence CE (Fig. 3f) well agrees with the field-enhancement driven emission at the third harmonic frequency. The CE value predicted at resonance is ~10^−5^, in agreement with the experimental results, confirming the improved accuracy of the adopted methodology (Fig. 3g).

### Second and third harmonic generation in (In_*x*_Bi_(1−*x*)_)_2_Se_3_/ Bi_2_Se_3_ heterostructures

The absence of SHG in the Bi_2_Se_3_ SSRR may be ascribed to the centrosymmetric nature of the Bi_2_Se_3_ bulk crystal dominating the optical response, combined with a lack of symmetry breaking in the TSS. More trivially, being a nonlinear mechanism, it could also be an effect of an inadequate electromagnetic coupling with the pump field. We thus investigated a set of Bi_2_Se_3_-heterostructures, comprising quintuple layers (QLs) of (In_x_Bi_(1−*x*)_)_2_Se_3_ adjacent to the Bi_2_Se_3_ slab^69^. In the first heterostructure (H1), a 20 nm thick Bi_2_Se_3_ layer was grown onto a 30 nm thick insulating (In_x_Bi_(1−*x*)_)_2_Se_3_ buffer layer, deposited onto a sapphire substrate. In a second sample (H2), the same (In_*x*_Bi_(1−*x*)_)_2_Se_3_ layer was grown on both sides of the Bi_2_Se_3_, resulting in an (In_*x*_Bi_(1−*x*)_)_2_Se_3_/Bi_2_Se_3_/(In_*x*_Bi_(1−*x*)_)_2_Se_3_ three-layered structure, a prototypical building block for vdW heterostructures, that could be repeated to give thicker, stacked MBE growth.

Micro-Raman spectroscopy experiments were performed to characterize the materials (see SI, Section S8). To shed light on the topological nature of the heterostructure-TIs, we also conducted low-temperature magneto-transport measurements. Both samples show resistances that decrease with temperature, indicating that scattering within these samples is dominated by electron-phonon interactions at high temperature (see SI, Section S9). The low temperature magnetoresistance also shows weak anti-localization at low field^70^, followed by a parabolic background in the longitudinal resistance which is indicative of ordinary magnetoresistance^70^. Furthermore, there is some slight non-linearity in the Hall coefficient, indicating the presence of multiple carrier species.

**Fig. 4:**
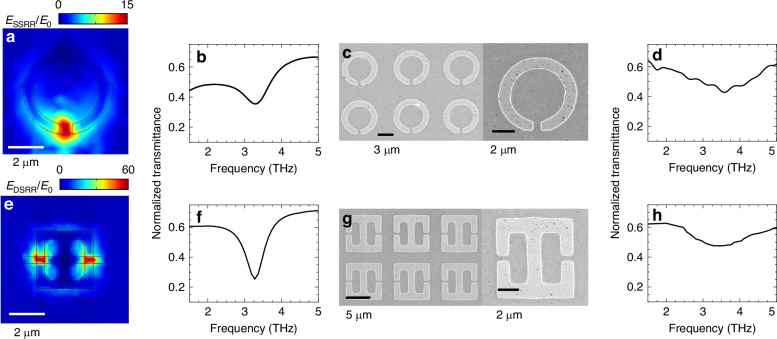
Ring resonators array embedding vdW topological insulator heterostructures. **a** Bi-dimensional map of the normalized electric field (*E*_SSRR_/*E*_0_) on the Bi_2_Se_3_ SSRR at the resonance frequency *ν*_SIM_ ~ 3.245 THz, calculated using FEM simulations on a SSRR fabricated on as-grown Bi_2_Se_3_ heterostructures. *E*_0_ is the electric field on the bare sapphire surface. **b** Simulated optical transmittance of the design shown in (**a**), extracted when the polarization of the input beam is parallel to the split gap dipole. **c** SEM images acquired on the SSRR fabricated on the (In_*x*_Bi_(1−*x*)_)_2_Se_3_/Bi_2_Se_3_/(In_*x*_Bi_(1−*x*)_)_2_Se_3_ sample, H2. **d** Experimental transmittance acquired on the sample shown in (**c**), using FTIR, under vacuum, with a spectral resolution of 1 cm^−1^, employing internal MIR (Globar) source and a helium-cooled Si bolometer. A rotating, wire-grid linear polarizer was positioned in front of the sample; the reported transmittance curve was obtained by taking the ratio of the curve acquired by selecting the linear polarization parallel to the split gap dipole (polarization angle 0°), with the one acquired at 90°, and then normalizing to account for the reflection losses of the sapphire substrate (~40%). **e** Normalized electric field (*E*_DSRR_/*E*_0_) map of the Bi_2_Se_3_ DSRR at the resonance frequency *ν*_SIM_ ~ 3.25 THz, calculated using FEM simulations for the array realized directly on the as-grown MBE Bi_2_Se_3_ sample. **f** Simulated optical transmittance of the design shown in (**e**), extracted when the input beam polarization is oriented parallel to the split gap’s dipole. **g** SEM images acquired on the DSRR fabricated on the (In_x_Bi_(1-*x*)_)_2_Se_3_/Bi_2_Se_3_/(In_*x*_Bi_(1−*x*)_)_2_Se_3_ sample, H2. **h** Experimental transmittance acquired on the sample shown in (**g**), using the same methods as (**d**)

**Fig. 5:**
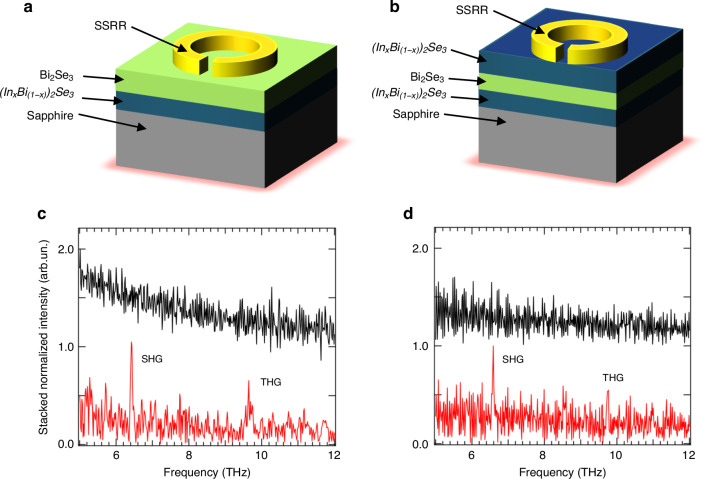
SHG and THG in vdW topological insulator metamaterials. **a** and **b** Schematic illustration of the single-SRR array embedding sample H1 (**a**) and H2 (**b**). **c** and **d** Emission spectra measured from the optically pumped samples **c** H1 and **d** H2, incorporating SSRR (black) and DSRR (red) design geometries, respectively. The spectra are measured in step-scan mode with a spectral resolution ~1 cm^−1^ and aperture size ~5 mm. The total light reflected by the samples was collected through the external input window of the FTIR spectrometer, with a silicon-bolometer connected to a lock-in amplifier for detection

The retrieved CE ~ 10^−5^ for THG on the single Bi_2_Se_3_ sample (Fig. 3) approached the signal-to-noise level reached in the pump and probe experiment. From the expected behavior of topological states in TIs, the second order nonlinearity, *χ*^(2)^ ≤ *χ*^(3)^, and so similar CE values are expected^44^ even for SHG in symmetry-breaking, pristine TIs. We, therefore, conceived an alternative resonator design to maximize the electric field amplification. This comprise a double split ring resonator (DSRR)^71^, realized by integrating, back to back, two split rings in one single resonator. A comparison between the single-, already tested, and the new double-SRR is shown in Fig. 4. To minimize any possible detrimental effects on the material quality associated with handling and processing, we opted for a fabrication approach that preserve the properties of the TI surface. Specifically, we patterned the resonator array directly on the as-grown MBE TI films, H1 and H2, and restricted the fabrication to one single step, i.e. without performing the etching step for the isolation of the active region around the split-gap amplification area (see schematics in Fig. 5a, b). First, we adjusted the resonator geometry for fabrication on a sapphire MBE substrate, while matching the pump frequency, via a simple scaling of the dimensions. During this design optimization, we adapted the model for the Bi_2_Se_3_ optical constants, discussed in Section 2, to match the conductivity of the heterostructures, using the parameters extracted from the magneto-transport experiment. As the field amplification is mostly connected to the architecture of the metallic micro-structured resonator, the design optimization procedure usually does not require the fine adjustment of the optical constants of the material, as long as any related possible shift in the resonance still matches the optical pump emission bandwidth. In fact, the presence of a trivial layer of (In_*x*_Bi_(1−*x*)_)_2_Se_3_ is only weakly affecting the resonance frequency and the field enhancement (see SI, Section S7). The resulting SSRR design (see Fig. 4a) has an overall smaller size, accounting for the higher real part of the sapphire refractive index *n*_Al2O3_ ~ 3.1 when compared to silicon oxide, *n*_SiO2_ ~ 2. More specifically, the sapphire substrate was modeled as a dielectric layer with non-zero real and imaginary optical constants, to account for the absorption in the THz and mid-IR ranges (see SI). At resonance, the field enhancement in the split gap area of the SSRR is comparable to the SSRR on SiO_2_ (see Fig. 4a), as expected in a simply scaled geometry, and the calculated transmittance shows a resonant absorption centered at *ν*_SIM_ ~ 3.25 THz and a *Q*_SIM_ ~ 3.3 (see Fig. 4b). The transmittance measured on the sample H2, after fabricating the SSRR resonator array using the scaled design (Fig. 4c), shows a resonance at a slightly higher frequency, *ν*_EXP_ ~ 3.52 THz, and a significantly broader width, corresponding to a *Q*_EXP_ ~ 1.53 (see Fig. 4d). The comparison between the calculated and experimental transmittances for the SSRR and the DSRR in Fig. 4 reveal, in both cases, a broader absorption and a blue shift of the resonance frequency in the experimental data. This behavior should be ascribed to optical losses and absorption paths not easily accounted in the numerical models, which inherently consider the systems as an ideal one in terms of, e.g., geometrical patterns, metallic layers, and absence of contaminations. On the other hand, the presence of an additional term in the optical conductivity ascribed to a free electrons-like surface layer, would lead to the same effect observed experimentally. Such resonant frequency discrepancies, particularly in the DSRR, implies a ~ 0.3 THz frequency mismatch between the resonator mode and the pump laser line, that here is partially compensated by the broader FWHM. However, the larger resonance width is not necessarily detrimental in the THG process, since the field enhancement is only weakly dependent on the *Q*-factor^31^. Indeed, while the *Q*-factor is a fundamental figure to ensure the matching between the resonator fundamental mode and the pump laser, a lower *Q*-factor is not necessarily implying a lower amplification. There are other factors lowering the *Q*-factor values, such as the array resonator-resonator cross-talking in dense arrays, that reduces the *Q*-value without affecting the field amplification.

We then tune the device geometry and assess the field enhancement in the DSRR (Fig. 4e, f). The field enhancement, extracted for an input beam polarization oriented parallel to the split-gap dipolar axis, reaches values of the order of ~10^2^ (Fig. 4e). The field amplification region is also extended well beyond the two split-gap areas. This behavior, together with the evidence that the electromagnetic field is mostly concentrated in a number of hot-spots that is twice the number of amplification spots formed in the SSRR, makes this design a more suitable candidate for the observation of nonlinear processes. The fabricated DSRR (Fig. 4g), realized on the same chip of the SSRR during the same fabrication run, shows a resonant-like transmittance (Fig. 4h) with a ~ 0.18 THz blue-shift and a lower *Q*-factor, if compared with the simulation (*ν*_SIM_ ~ 3.26 THz and *Q*_SIM_ ~ 3.1). Specifically, ν_EXP_ ~ 3.44 ± 0.4 THz and *Q*_EXP_ ~ 2.02 ± 0.45, exhibiting an overall behavior similar to that measured on the SSRR. Similar results were found for the transmittance measured on the sample H1.

To assess the non-linear origin of any possible signal emitted by the optically pumped heterostructure SRR array, we initially measure the signal detected by a Si bolometer after filtering the QCL emitted frequency with a 4 mm-thick Ta-filter. To investigate possible SHG or THG in the Bi_2_Se_3_-heterostructure SRR arrays, we used the external-pump apparatus of Fig. 2a, but slightly modified to collect the beam reflected by the TI surface, avoiding higher harmonic absorption by the sapphire substrate. This was achieved by mounting the TI film on a rotating stage set at a 45° angle with respect to both the incoming beam angle, and to the collection axis of the FTIR spectrometer. Under these conditions, the beam emitted by the QCL is focused onto the sample surface, while the reflected beam, comprising the linear reflection and the nonlinear emission terms, is collected at a 90° angle with respect to the incidence beam, and directly fed onto the FTIR spectrometer. The emission spectra are then retrieved by measuring the total signal collected from the illuminated Bi_2_Se_3_ -SRRs, after filtering out the fundamental mode (QCL pumping frequency).

The emission spectra are reported in Fig. 5c, d, for sample H1 (Fig. 5c) and H2 (Fig. 5d), respectively, as acquired for the SSRR (black) and DSRR (red) resonators. Remarkably, we retrieved clear SHG peaks and slightly weaker THG peaks in both H1 and H2 for the DSRR array, i.e. the higher field enhancement design, meaning that, independently from the nature of the TSSs (trivial or topologically protected) SHG is activated but with a stronger bulk contribution, inducing THG in the sample with no topological protection (H1). No evidence of either SHG or THG was seen in the SSRRs, likely due to the fact that the field enhancement is not strong enough to allow a conversion efficiency within the noise limits of our set-up.

## Discussion

The conversion efficiencies, determined from the experimental data, are listed in Table 1, together with the THG efficiency, extracted from the previously discussed Bi_2_Se_3_ SSRR. The THG signal, retrieved on both the H1 and H2 DSRRs, are in broad agreement with the CE values found on the simple Bi_2_Se_3_ SSRR transferred on SiO_2_/Si, testifying a similar origin of the nonlinear process. The presence of a THG signal that is less intense then SHG, indicates a lower third-order conversion efficiency. This is in apparent contrast with other reports^44^, and with the general property of stronger odd nonlinearities in centrosymmetric crystals^48^. Our experimental findings suggest the existence of alternative mechanisms supporting the SHG, such as IR-active phonon assisted modes^72^ superimposed to the resonator-assisted complex optical response, that may increase even-order nonlinearities. However, it should be noted that the decoding of individual harmonics in the interferometric trace, and the procedures used to extract the spectra via Fourier transforms in the frequency domain, is not trivial, and could be masked in the presence of a dominant noise.

**Table 1:** Experimental conversion efficiencies

Sample	SHG	THG
H1, DSRR	(3.5 ± 1) × 10^−5^	(2.7 ± 0.7) × 10^−5^
H2, DSRR	(2.5 ± 1) × 10^−5^	(1.9 ± 0.7) × 10^−5^
A, SSRR	Not detected	(1.3 ± 0.3) × 10^−5^

From the measured experimental conversion efficiencies we have extracted the third order non-linear susceptibility *χ*^3^, as follows. The conversion from the pump frequency to the TH order is described by the relation^14^: ${{E}}_{{\rm{TH}}}={\rm{\gamma }}{L}{{E}}_{0}^{3}$, where *E*_0_ ~ 8.7 × 10^4^ V/m is the pump field at the fundamental frequency, *E*_TH_ is the up-converted field, *L* = 2 nm is the TI effective thickness, and *γ* is related to *χ*^3^ by the equation^14^:

$${{\chi }}^{3}=\frac{{\gamma }}{3/4\left({\pi }{{\nu }}_{{\rm{TH}}}/{c}{{n}}_{{\rm{TH}}}\right)}$$

where *c* is the speed of light, *ν*_TH_ = 9.63 THz is the TH frequency, and *n*_TH_ ~ 1.8 is the TI refractive index at the TH. By estimating the electric field at the TH frequency from the experimental CE, we obtain the *χ*^3^ values reported in Table 2.

**Table 2:** Third order non-linear susceptibility

Sample	*χ*^3^ (m^2^ V^−2^)
H1	9.2 × 10^−9^
H2	6.9 × 10^−9^
A	3.31 × 10^−9^

The extrapolated *χ*^3^ values, referring to the combined TI/resonator system, are of the same order of magnitude than those retrieved in graphene, *χ*^(3)^ ~ 10^−9^ m^2^/V^2^, i.e. almost 7 orders of magnitude larger than THz QCL semiconductor heterostructures (*χ*^(3)^ ~ 7 × 10^−16^ m^2^/V^2^)^19,20^.

In conclusion, we designed a Bi_2_Se_3_-embedded SRR to induce field amplification in the split-gap region of a circularly shaped-ring, tuned to match the frequency of the external pump laser. THG at 9.63 THz from a ~20 thick Bi_2_Se_3_ layer was retrieved, at a frequency corresponding to the third order frequency up-conversion of the fundamental pumping laser mode. This arises from the film’s nonlinear response, when dominated by the centrosymmetric properties of the Bi_2_Se_3_ crystal. SHG and THG at 6.42 THz and 9.63 THz, respectively, are revealed by embedding vdW (In_x_Bi_(1−*x*)_)_2_Se_3_/Bi_2_Se_3_-heterostructures into DSSRs, which induce a two order-of-magnitude field enhancement factor. The improved film quality in the TI heterostructure led to a strong nonlinear response, mainly provided by the TSSs, which induce symmetry breaking and unlock even-order nonlinear processes. Our experiments demonstrate a novel pathway for light generation in a frequency range between the mid-IR and THz regions, i.e. 25–60 μm (12–5 THz), traditionally difficult to be exploited with practical solid-state laser technologies, owing to parasitic optical phonon absorption (the *Restrahlenband*) in the constituent III–V materials used to fabricate the only miniaturized optical source adopted in this range, the QCLs. On-chip integration of the explored novel material systems with monolithic QCL sources, can be a very promising route to maximize the conversion efficiency of both THG and SHG at high (>6 THz) THz frequencies, exploiting the huge intracavity QCL power. The generation of coherent light in the 6–12 THz range, may indeed have great impact on the development of photonic sources, capable to unlock a plethora of disruptive applications, nowdays seriously hindered by the lack of compact solid state technologies, such as vibrational molecular spectroscopy of hydrocarbons^73^, providing enabling technologies in many fields, such as quantum optics^74,75^, and nano-spectroscopy^76^. Finally, we show here that playing with the TI material systems, we can induce symmetry breaking effects that can trigger, by design, odd- or combined even and odd-orders frequency up-conversion in the THz frequency range.

## Materials and methods

### Bi_2_Se_3_ based heterostructures: growth and characterization

The samples were grown on [0001] sapphire substrates. Following ref.^69^, the initial (In_*x*_Bi_(1−*x*)_)_2_Se_3_ layer was nucleated by depositing 5 nm of Bi_2_Se_3_ at 235 °C (temperatures measured by a thermocouple attached to the sample manipulator), followed by 5 nm of In_2_Se_3_, and then annealing the stack at 325 C under Se flux to create a 10 nm-thick (In_*x*_Bi_(1−*x*)_)_2_Se_3_.layer. After annealing, sample H1 was cooled under a Se flux and an additional 20 nm of (In_*x*_Bi_(1−*x*)_)_2_Se_3_ was deposited, for a 30 nm-thick total layer, followed by a single, 20 nm-thick Bi_2_Se_3_ layer. For sample H2, a similar Bi_2_Se_3_ layer was grown, then capped with 20 nm (In_*x*_Bi_(1−*x*)_)_2_Se_3_. Both samples were then diced and patterned into standard hall bars by chemical wet etching, and Cr/Au ohmic contacts were subsequently deposited before being loaded into a continuous flow helium cryostat with a base temperature of 1.6 K and a 8 T superconducting magnet. Transverse and Hall conductivities were measured using standard lock-in amplifier techniques at a frequency of 199.77 Hz.

### Nanofabrication of the Bi_2_Se_3_ resonator

The Bi_2_Se_3_ resonators were fabricated on a high (10^4^ Ω cm)-resistivity 300-mm-thick Si substrate, coated with a 300 nm-thick SiO_2_ layer (by Siltronyx). The CSRR array pattern is defined by optical lithography using a LOR3A/S1805 bilayer photoresist, on a 5 × 5 mm^2^ area, followed by metal evaporation and liftoff of 10 nm/100 nm of Cr/Au. The 1×1 cm^2^ Bi_2_Se_3_ film is then transferred onto the top of the SSRR array, following a PMMA-assisted method^57^, using a 30% KOH solution at ~70 °C to promote the delamination of the MBE grown TI from the Sapphire substrate. The Bi_2_Se_3_ in the gap area is then patterned with a second lithographic step, followed by a plasma-Ar etching to remove the TI film from the area surrounding the gap, with a final cleaning by acetone soaking.

The resonators embedding the vdW TI heterostructures are fabricated by electron beam pattering directly on the as-grown MBE samples, employing the undercut-profile PMMA 950K polymers as a liftoff e-beam resist.

### Numerical simulations

The SSRR single unit element (Fig. 1b), comprises a perfect electric conductor (PEC) C-shaped ribbon (external radius, 4.1 µm, internal radius 3.2 µm, gap 1.5 µm) at the interface between a 300-nm-SiO_2_/Si bottom and air top domains. The unit cell pitch is *p* = 14.5 μm, and the total height of the calculation domain is 85 μm, i.e. 35 μm-thick dielectric substrate and 50 μm-thick volume of air. The FEM calculation (by COMSOL Multiphysics) were carried out by setting Floquet boundary conditions along the *x*,*y* side edges, and scattering boundary conditions on the top and bottom domain edges (*z* edges). The transmittance is calculated as ${T=\left|{S}_{21}\right|}^{2}$, where the *S*-parameters are extracted by placing one input port on top of the air domain and one receiver port on the bottom of the Si domain. A rectangular 5 × 5 µm^2^ area around the split gap is defined, and a transition boundary condition is set, based on the Bi_2_Se_3_ complex optical conductivity, calculated from the procedure described in the text, and reported in the SI. *A*_G_ is calculated as the ratio between the electric field magnitude, averaged over the gap region surface, and the average field magnitude computed in absence of the resonator, from the same simulation module, eliminating the metal split ring. In the Bi_2_Se_3_-heterostructure SSRR and DSSR, the Bi_2_Se_3_ film, modeled with the same complex optical constant, was set as transition boundary layer over the entire region surrounding the metal ring.

### Optical Set-up

The laser used in this work is a QCL emitting at 3.25 THz (*λ* = 108 µm), based on a bound to continuum-optical phonon hybrid active region design^77^. The 25-µm-thick GaAa/AlGaAs active region is patterned into a surface-plasmon waveguide onto a semi insulating GaAs substrate, using a combination of wet etching and metal deposition. A 5-nm-thick, 40-µm-wide Ni side-absorber, overlapping on 3 µm on each side of the upper 150 nm-thick Au over-layer, is introduced on each edge of the ridge to increase the difference in losses between fundamental and higher order transverse modes, in order to suppress fully the higher-order competing modes. The 700-nm-thick heavily Si-doped (5 × 10^18^ cm^−3^) GaAs top contact layer lies between the active region and the substrate.

The QCL, driven in pulsed-mode (1 µs pulse, 50 kHz repetition rate) and emitting 2.5 W peak power is focused onto a ~ 0.4 mm spot on the surface of the Bi_2_Se_3_-resonators array facing the external-input window of an FTIR spectrometer (Bruker Vertex V80). The THG or SHG signal is isolated after filtering the QCL emission with a 6 THz high bandpass, 2 mm-thick, Ta-filter. The spectra are acquired under vacuum, in step-scan mode, with a resolution of 1 cm^−1^. The signal is measured by a helium-cooled Si-bolometer, connected to a lock-in amplifier, referenced to an amplitude modulated signal at 317 Hz, that is also simultaneously gating the QCL bias pulses.

## Supplementary information

Supporting Information

## Data Availability

The data presented in this study are available on reasonable request from the corresponding author.

## Code availability

The relevant computer codes supporting this study are available from the authors upon reasonable request.
