# Supplementary material for: Second and third harmonic generation in topological insulator-based van der Waals metamaterials
Source: Light Sci Appl. 2025 Sep 22;14:337. doi: 10.1038/s41377-025-01847-5 (PMC12454650; doi:10.1038/s41377-025-01847-5)
Supplement: Supplementary file 1 — Supporting Information [file 41377_2025_1847_MOESM1_ESM.docx]

**Second and third harmonic generation in topological insulator-based van der Waals metamaterials**

Alessandra Di Gaspare^1^, Sara Ghayeb,^1^ Craig Knox^2^, Ahmet Yagmur^3^, Satoshi Sasaki^3^, Mohammed Salih^2^, Lianhe Li^2^, Edmund H. Linfield^2^, Joshua Freeman,^2^ and Miriam S. Vitiello^1^

^1^NEST, CNR-NANO and Scuola Normale Superiore, 56127, Pisa, Italy

*^2^School* *of* *Electronic* *and* *Electrical* *Engineering*, *University* *of* *Leeds*, *Leeds*, *LS2* *9JT*, *UK*

*^3^School* *of* *Physics* *and* *Astronomy*, *University* *of* *Leeds*, *Leeds*, *LS2* *9JT*, *UK*

**S1. Bi_2_Se_3_ optical constant**

The ~20nm thick Bi_2_Se_3_ film of Section 2-3 of the main article has been characterized by low-temperature magneto transport^1^. The fitting analysis of the experimental magnetoresistance curves reveal the presence of two surface carrier bands, the first having lower mobility and density of carriers, attributed to the trivial surface stated (2D massive electrons) and the second associated with the topological surface states. Each one of these two band can be treated independently, providing its own contribution to the optical conductivity, namely:

$\sigma_{DM}\left( \nu\right)=\frac{-iD_{0}}{\pi}\frac{1}{\left( 2\pi\nu+i\Gamma_{0} \right)}$ (S1)

$\sigma_{DM}\left( \nu\right)$is here the intraband, Drude-like complex conductivity of the Bi_2_Se_3_ topological Dirac material (DM) forming the surface state, where $D_{0}={E_{F}e^{2}}/{\hbar^{2}}$ is the linear Drude weight, E_F_ the Fermi energy, *e* is the electron charge, *ħ* is the reduced Planck constant, $\Gamma_{0}={\tau_{0}^{-1}=e{v_{F}}^{2}}/{E_{F}\mu}$ is the scattering rate, v_F_ the Fermi velocity, and µ is the carrier mobility. For the Bi_2_Se_3_ topological state, we set v_F_ = 5×10^5^ms^-1^, $E_{F}=\hbar v_{F}\sqrt{4\pi n_{DM}}$, with *n*_DM_ the carrier density of the topological surface state. From the magnetotransport measurements, we extract the values µ=1000 cm^2^/V^-1^s^-1^, and *n*_DM_=0.65×10^13^cm^-2^, and we obtain E_F_ = 296meV.

$\sigma_{2DEG}\left( \nu\right)=\frac{i}{2\pi\nu}\frac{{n_{2DEG}e}^{2}}{m^{*}}$ (S2)

$\sigma_{2DEG}\left( \nu\right)$is the complex conductivity of the 2D classical electron gas, where m*=0.15m_e_ in Bi_2_Se_3_ trivial surface state, and n_2DEG_ = 1.9×10^13^cm^-2^ is 2D carrier density extracted from the massive electron band contribution to the Hall magnetoresistance. The surface state contribute to the total permittivity is then:

$\varepsilon_{2D,tot}=1+2\times\left( \frac{{\sigma_{DM}+\sigma}_{2DEG}}{2\pi\nu\varepsilon_{0}t_{TI}} \right)$ (S3)

where the factor 2 accounts for the topological surfaces on both sides of the Bi_2_Se_3_ slab, ɛ_0_=8.85×10^-12^ Fm^-1^ is the vacuum permittivity, and t_TI_ ~ 1nm is the quantum layer thickness of the Bi_2_Se_3_ surface state.

The 20 nm thick bulk has a permittivity given by the sum of the Drude free carrier primitivity, $\varepsilon_{Drude},$and three Lorenz-Drude terms arising from the E_u_ and A_2u_ TO phonons, and the bandgap absorption oscillator^1,2^:

$\varepsilon_{Drude}= \frac{\omega_{Drude}^{2}}{\omega^{2}+i\omega\gamma_{Drude}}$ (S4)

$\varepsilon_{Bulk}=\varepsilon_{Drude}+\sum_{j=1}^{3} \frac{\omega_{pj}^{2}}{\omega_{0j}^{2}-\omega^{2}-i\omega\gamma_{j}}$ (S5)

Table 1 displays all the parameters appearing in eqs.S4-S5.

| **Parameter** | **Value** | **Description** |
| --- | --- | --- |
| ω_Drude_ | 900 cm^-1^ | Free carrier plasma frequency BiSe bulk |
| γ_Drude_ | 733 cm^-1^ | Drude damping rate |
| ω_p1_ | 675.9 cm^-1^ | TO phonon (E_u_) oscillator strength |
| ω_01_ | 63.03 cm^-1^ | TO phonon frequency |
| γ_01_ | 17.05 cm^-1^ | TO phonon damping rate |
| ω_p2_ | 100 cm^-1^ | TO phonon (A_2u_) oscillator strength |
| ω_02_ | 127 cm^-1^ | TO phonon frequency |
| γ_02_ | 10 cm^-1^ | TO phonon damping rate |
| ω_p3_ | 11249 cm^-1^ | bandgap oscillator strength |
| ω_03_ | 2029.5 cm^-1^ | bandgap frequency |
| γ_03_ | 117.3 cm^-1^ | bandgap damping rate |

**Table 1.** Optical parameters of BiSe^1,2^

The Bi_2_Se_3_ total permittivity is then $\varepsilon_{BiSe}=\varepsilon_{Bulk}+\varepsilon_{2D}.$

The complex refractive index (n,k) is then extracted from the relationships:

$n=Re\left( \sqrt{\varepsilon_{BiSe}} \right)$; $k=Im\left( \sqrt{\varepsilon_{BiSe}} \right)$ (S6)

****The real and imaginary parts of the complex refractive index are reported in Fig. S1a. In Fig.S1b, is shown only the contribution of the bulk, that will be used in the third harmonic generation numerical model, to account for the bise nonlinear response from the surface states, with the Bi_2_Se_3_ bulk providing the dielectric environment for the nonlinear, surface current generator.

**Figure S1**. a) Complex refractive index of the Bi_2_Se_3_ film, calculated from the total permittivity, comprising the surface states and the bulk contributions. b) Complex refractive index of the Bi_2_Se_3_ bulk.

**S2. Effect of the TI film bending**

The large area Bi_2_Se_3_ film is robust after the wet chemical detaching, and upon transfer the damage or bending is usually negligible. However, to evaluate any possible influence on the resonator optical properties that could affect its response, in Fig.S2 (top panel) we compare the simulations performed by considering Bi_2_Se_3_ as suspended and with a slightly bent surface across the two metal edges of the resonator gap, modeled as 100nm thick Au (optical properties defined in COMSOL library), with the case of a Bi_2_Se_3_ film in contact with the substrate below (bottom panel). Any significant difference is visible in the two cases.

**Figure S2:** Electric field distribution across the gap area of the Bi_2_Se_3_-SSRR, calculated using FEM simulations in the two cases: suspended film (top), and in contact with the substrate (bottom)

**S3. Single split ring resonator: field amplification**

The response of the single split ring resonator (SSRR) of Section 2-3, when illuminated by a plane wave linearly polarized along the direction indicated by the arrow in Fig.1b, leads to the formation of a hot spot, i.e. a region where the electromagnetic field intensity is up to two orders of magnitude higher than the field of the incoming beam. The resonator essentially provides a field enhancement, at the resonance, in the split gap area, owing to the dipolar mode established in the region enclosed by the two metal arms. The resonance frequency and the field enhancement can be tuned, by design, through the ring geometry; however, they are also affected by the dielectric response of the split gap region. To account for the presence of the thin Bi_2_Se_3_ layer, and to highlight its role in determining the field amplification needed to enhance the frequency up-conversion efficiency, in Fig.S3 we compare the gap amplification simulated in the bare SSRR (black) and in the SSRR with Bi_2_Se_3_ in the gap (red). The bare SSRR was realized by the metal ring, modeled as a perfect electric conductor (PEC), on top of a 300nm-SiO_2_/Si substrate, shaped as a THz-transparent dielectric material (n_SiO2_~2, n_Si_ ~3.4). The Bi_2_Se_3_ integrated SSRR comprises a 5×5µm^2^ area around the gap, set as a transition boundary condition with the complex refractive index introduced in Section S1.

The gap amplification peak is slightly blue-shifted and decreased in the BiSe/SSRR, as a consequence of the different optical contrast between the materials of the resonators, particularly localized in the split gap, i.e. the most sensitive area to electromagnetic changes.

**Figure S3**. Comparison of the field amplification gap simulated on the bare SSRR (black) and in the Bi_2_Se_3_/SSRR, with the Bi_2_Se_3_covering the 5×5µm^2^ area around the gap area, described as a transition optical layer with n,k shown in Fig. S1.

**S4. Hot electron nonlinear response in Bi_2_Se_3_**

In the THz range, the optical response of the Bi_2_Se_3_ is ruled by the intraband absorption^3^. The presence of a high-power optical beam drives the system to a non-equilibrium state with an excess distribution of carriers at the energy of the optical pump. This leads to the nonlinear response of the system^4^, that is then dominated by the thermal electrodynamic processes resulting in the Dirac carriers, on a timescale comparable to the light oscillations, as reported for single layer graphene^5,6,7,8^. At first ultrafast (~20fs^4^) carrier-carrier scattering determines the initial energy redistribution, bringing the system into a non-equilibrium state with electrons sharing an hot-electron temperature T_e_.^6^ Then, the system relaxes back to an equilibrium state^7,8^, depending on the relaxation cooling channels available. In the present work, the pump source varies on a timescale (~µs pulse duration, 50kHz repetition rate) much longer than any possible relaxation channel, leading the system into a steady excitation state at T_e_ is realized^9,10^, where:

$T_{e}=T_{\mathrm{sub}}+\frac{P_{\mathrm{in}}\tau_{\mathrm{cool}}}{C_{e}}$ (S7)

with T_sub_=300K is the temperature at equilibrium, *P*_in_ is the excitation power intensity, in units of Wcm^-2^, and τ_cool_ is the cooling time. We express the heat capacitance, C_e_, as in the case of single layer graphene, for the limited doping regime (E_F_ *<<* K_B_T_e_) as^9^:

$C_{e,doped}=\frac{2\pi E_{F}}{3\left( \hbar v_{F} \right)^{2}}k_{B}^{2}T_{e}$ (S8)

****We assume *τ*_cool_=3.5ps^11^, and by combining Eqs. S7 and S8, we can calculated the T_e_ dependence on the pump power density (Fig. S4), by using for the BiSe film, v_F_ =5×10^5^ ms^-1^ and E_F_ =290meV.^1^

**Figure S4:** Hot-electron temperature in the Bi_2_Se_3_ pumped optically with a pump beam focused on a 0.7 mm spot, as a function of the power, according to Eqs. S6-7.

**S5. Third-order field-dependent conductivity**

In presence of an intense excitation beam, the SLG nonlinear response can be expressed through a field-dependent conductivity^10^:

$\sigma_{\mathrm{tot}}\left( \nu\right)=\sigma_{0}\left( \nu\right)+\left| E_{0}\left( \nu\right) \right|^{2}\sigma_{3}\left( \nu\right)$ (S9)

where *E*_0_ is the field intensity, and $\sigma_{0}\left( \nu\right)$ is the Bi_2_Se_3_linear total conductivity is calculated in Section S1. The nonlinear term of the conductivity, σ_3_, is the Kerr conductivity^6^:

$\sigma_{3}\left( \nu\right)=\eta\left[ \sigma_{\mathrm{Kerr}}\left( \nu\right) \right]$ (S10)

whose numerical expressions are^12^:

$\sigma_{\mathrm{Kerr}}\left( \nu=v_{\mathrm{eff}} \right)=\frac{i9e^{6}v_{F}}{4\pi\hbar^{4}}\frac{D_{\mathrm{he}}}{\left( 2\pi\nu+i\Gamma_{\mathrm{he}} \right)\left( -2\pi\nu+i\Gamma_{\mathrm{he}} \right)\left( 4\pi\nu+i\Gamma_{\mathrm{he}} \right)}$ (S11)

where the substitution of $\nu$ with $\nu_{\mathrm{eff}}=\left( \nu^{2}-\nu_{0}^{2} \right)/\nu$ accounts for the plasmonic LC resonance of the SRR, and $\eta\sim0.6$ is the total filling factor of SRR array. The *E*_F_-dependent parameters D_he_ and Γ_he_ are the hot electron Drude weight and scattering rate, can be written as^13,12^:

$D_{\mathrm{he}}=D_{0}\left[ 1-\frac{1}{6}\left( \frac{\pi k_{B}}{E_{F}} \right)^{2}{T_{e}}^{2} \right]$ (S12)

$\Gamma_{\mathrm{he}}=\Gamma_{0}\left[ 1+\frac{1}{6}\left( \frac{\pi k_{B}}{E_{F}} \right)^{2}{T_{e}}^{2} \right]$ (S13)

The hot electron optical response is then captured by the transmittance, as for Eq.1 of the main text.

**S6. Refined numerical model for third harmonic generation (THG)**

In the refined model, the THG conversion efficiency (CE) is extracted by setting up 2D simulations of the SRR array, defining the unit cell with the same geometrical configuration used for the extraction linear parameters (resonance frequency, Q-factor and field amplification) presented in Section 2 of the main article, and using frequency domain interface in (COMSOL Multiphysics Inc). A perfectly matched layer (PML) boundary condition in used in the *z* direction defined in Fig.3d (manuscript) while periodic boundary condition was applied along the *x,y* directions. The Bi_2_Se_3_ is included by defining a 20-nm thick layer with the n,k shown in Fig.S1b, i.e. modeling the Bi_2_Se_3_ insulating bulk, with two surface current density generators on both the top Bi_2_Se_3_/air and the bottom Bi_2_Se_3_/SiO_2_ interfaces. The current generators are first used to generate the linear electric field E_FH_, relying on the linear conductivity

$J_{0}=\sigma_{0}E_{\mathrm{FH}}$ (S14)

then, during a second simulation iteration, they generate the third harmonic field E_TH_, relying on the field-dependent Kerr conductivity of Eq.S9, from the following equations S15-16:

$J_{\mathrm{tot}}=\sigma_{0}E_{\mathrm{TH}}+J_{3}$ (S15)

where

$J_{3}\left( \nu\right)=\sigma_{3}\left( \nu\right)\left[ 2\left| E_{FH}\left( \nu\right) \right|^{2}E_{FH}\left( \nu\right)+c.c. \right]/3$ (S16)

To calculate the CE associated to THG, we set an incident plane wave with transverse magnetic (TM) polarization and input power density I_0_ (Wm^-2^), irradiating the resonator plane. This is corresponding to an equivalent electric field: $E=\sqrt{Z_{0}I_{0}}$, where $Z_{0}$ = 377Ω is the vacuum impednacne, and $I_{0}$ is set in agreement with the relation: $I_{0}=\frac{P_{\mathrm{in}}}{A\mathrm{spot}}$ , with A_spot_ ~0.35mm^2^ is the illuminated spot area. The TH signal is generated by the Bi_2_Se_3_ embedded in the SRR, and irradiated back in the free space. CE is calculated as P_TH_/P_in_ where P_TH_ is the power outflow of the TH wave, calculated by the module of the Poynting vector of the TH wave.

**S7. (In_x_Bi_(1-x)_)_2_Se_3_/Bi_2_Se_3_and Sapphire optical constants**

The field amplification in the split gap of the resonator is only partially sensitive to the presence of the integrated TI film, as it is mainly dependent on the architecture of the metallic micro-structured resonators (Fig. S5b,c). We first consider the optical conductivity of the (In_x_Bi_(1-x)_)_2_Se_3_/Bi_2_Se_3_ film (In contend x=0.5), by adding a Drude-like term to the complex permittivity, accounting for bi dimensional electron gas in the **(**In_x_Bi_(1-x)_)_2_Se_3_ and by scaling the bulk contribution following the parameters in ref.^14^

$\varepsilon\left( v \right)=\varepsilon_{\infty}\left( 1-\frac{\nu_{Drude}^{2}}{\nu^{2}+i\gamma_{Drude}\nu} \right)$ (eq.S17)

The list of parameters used for the calculation is shown in table 2, and the as calculated complex refractive index in the frequency range of interest is shown in Fig.S5a.

| **Parameter** | **Value** | **Description** |
| --- | --- | --- |
| ν_Drude_ | 2.64 THz | Free carrier plasma frequency BiSe bulk |
| γ_Drude_ | 14THz | Drude damping rate |
| ε∞ | 18.8 | High frequency permittivity |

**Table 2.** Optical parameters of **(In_x_Bi_(1-x)_)_2_Se_3_** (from ref.^14^)

**Figure S5:** a) Complex refractive index calculated for the sample H1 (In content x=0.5). b) 2D map of the field amplification at the resonance, calculated for the DSRR c) Field amplification extracted from the simulation of the DSRR, performed considering the DSRR coated with a transition boundary condition having the complex refractive index of the Bi_2_Se_3_-only shown in Fig.S1a (red) and In_x_Bi_(1-x)_)_2_Se_3_/Bi_2_Se_3_ heterostructure shown in (a) (black).

**S8. Sapphire optical constants**

The response of the split ring resonators realized on the as-grown complex TI heterostructure samples of is affected by the different dielectric substrate. The higher sapphire refractive index n_AlO_~3.1^15^, if compared with the n_SiO2_, implies a rescaling of the geometry such that the ring geometry needs to be roughly ~ n_AlO_/ n_SiO2_ smaller to match the same frequency of the pump laser. Moreover, the sapphire absorption at higher frequency must be considered, particularly in the prediction of the harmonic generation conversion efficiency. We model the absorption of the Sapphire by following the ref.^15^, and generate a numerical absorption coefficient α[cm^-1^], in quantitative agreement with their measured behavior. Then we set a frequency independent real part of the refractive index at n_AlO_~3.1, and we extract the imaginary part from $k={\alpha c}/{4\pi\nu}$, where c is the speed of light. The results of such model are shown in Fig.S6a, reporting the absorption, and Fig.S6b, reporting the refractive index, in the frequency range of interest in the present work.

**Figure S6**: **(a)** Absorption coefficient of the Sapphire substrate in the frequency range of interest. **(b)** Complex refractive index of the Sapphire.

**S8. Micro Raman in (In_x_Bi_(1-x)_)_2_Se_3_/ Bi_2_Se_3_ heterostructures**

Micro-Raman spectroscopy experiments were performed to characterize the as grown heterostructures embedded in sample H1 and H2. Raman spectra (Horiba, Explora Plus microscope) were measured using a 532nm laser delivering 1 mW optical power, focused to a ~2µm spot. In the first case (Fig.S7a, H1), the E_g_^1^ A_1g_^1^, A_1g_^2^ and E_g_^2^ Raman modes of the Bi_2_Se_3_ are observed at wavenumbers of 33.1, 66.3, 170.4 and 127.0cm^-1^, respectively. For H2 (Fig.S7b), the picture is less trivial. The foremost Raman peaks of the Bi_2_Se_3_ are still visible, albeit over a significant, broad band background: E_g_^1^ A_1g_^1^, A_1g_^2^ and E_g_^2^ sit at 30.2, 65.9, 165.9 and 127.6cm^-1^, respectively. Three weaker peaks at 76.4, 159 and 182.3cm^-1^ are also detected, indicating the presence of weaker vibrational modes. The lower energy peaks may reflect the IR-active modes (E_u_ and A_1u_) of the Bi_2_Se_3_^16^, or they may be replicas of the A_1g_^1^ and the A_1g_^2^ peaks, related to the (In_x_Bi_(1-x)_)_2_Se_3_ layer or the (In_x_Bi_(1-x)_)_2_Se_3_/Bi_2_Se_3_ heterointerface in sample H2.

**Figure S7**: **(a-b)** Micro-Raman spectra for the as grown Bi_2_Se_3_-heterostructures (a) H1and (b) H2.

**S9. Magneto-spectroscopy**

To shed light on the topological nature of the heterostructure-TIs, we conducted low-temperature magneto-transport measurements. Both samples show resistances that decrease on cooling, indicating that scattering within these samples is dominated by electron-phonon interactions at high temperature. The low temperature magnetoresistance shows weak anti-localization at low field and a parabolic background in the longitudinal resistance (Fig.S8a), indicative of ordinary magnetoresistance^17^. A slight non-linearity in retrieved in the Hall coefficient (Fig.S8b), indicating the presence of multiple carrier species.

The origin of the carriers was further explored by examining the Shubnikov de-Haas (SdH) oscillations within the magnetoresistence^18^. To isolate the oscillations, the data was first smoothed with a Savitzky–Golay filter, using a 3^rd^ order polynomial fit, and taking the second derivative to remove any smoothly varying background. The results of this procedure are shown in Figure S8c. Both samples show oscillations above 5T (< 0.2T^-1^) that are periodic in inverse magnetic field, with the same periodicity of 46±1T, corresponding to a carrier density of (2.2 ± 0.1)×10^12^cm^-2^. The oscillations retrieved above 0.3 T^-1^ are not periodic and may, in fact, arise from universal conductance fluctuations. We then assign each periodic peak in Fig.S6c, i.e. troughs in the magneto-resistance, to filled Landau levels, and construct the Landau Fan diagram shown in Fig.S8d. Due to the Berry curvature, carriers orbiting around a Dirac cone, such as those that occupy topological surface states, will pick up an extra π phase when compared to conventional carriers^18^. As such the y-intercept of a Landau fan diagram will show whether an oscillation arises from topologically trivial (an intercept of ±1 or 0) or non-trivial (±0.5) carriers. We find that, while both sets of SdH oscillations arise from a similar carrier density, the intercept of the Landau fan diagram for sample H1 is 0±0.3, whereas the intercept for sample H2 is 0.6±0.2, indicating that the oscillations in the former probably arise from topologically trivial transport, possibly due to band bending at the TI-vacuum interface, while the carriers in sample H2 are topologically protected.

**Figure S8**: **(a-b)** (a) Magnetoresistance and (b) Hall resistance of samples H1 and H2; **(c)** 2^nd^-order differential of the data in (a), plotted as a function of inverse field, showing the presence of SdH oscillations. **(d)** Landau fan diagram constructed from the data in (c), of sample H1 (black) and H2 (red).

**S10. Transmittance of the bare resonator-array transmittance**

Figure S9 shows the transmittances measured on the single SRR array (SSRR, black) and the double SRR (DSRR, red) realized on bare sapphire substrate. The transmittance are measured by mounting the samples in the internal compartment of the FTIR, probed with the internal source of an FTIR spectrometer (Globar), under vacuum, in rapid scan mode (spectral resolution 1 cm^-1^), and placing a wire-grid polarized in front of the array so to select the polarization-active direction and the not active polarization at 90 degrees, where the frequency transmitted is flat. The SSRR shows a resonance ν_SSRR_ ~ 3.17THz and a Q-factor Q_SSRR_ ~5, comparable with the BiSe integrated array. For the DSRR, the corresponding values are ν_DSRR_ ~ 3.14THz and Q_DSRR_ ~3.2.

**Figure S9:** FTIR transmittance measured on the SSRR (black) and on the DSRR (red), extracted normalizing the sample transmitted signal measured when the array was illuminated by a broadband source (Globar), after filtering the linear polarization parallel to the ring dipole, with the signal acquired with the polarizer filter set at 90°.

**Section S11. Dependence of the nonlinear up-converted signal on the pumping power**

We measure the total signal detected by the Si bolometer, after filtering out the QCL fundamental mode with a 1-mm thick Ta filter, and while keeping the spectrometer moving mirror at a fixed position, on the sample A (Fig.S10a) and H1 (Fig.S10b), as a function of the power of the QCL, varied by changing the driving current, in the reasonable range in which a detectable signal can be retrieved (1.8-2.5W). The results agree with a polynomial fit accounting for a purely 3^rd^ order power law (S10a), for the sample A, while contains both a 2^nd^ and 3^rd^ order power law in panel b, retrieved on sample H1(S8b). In this latter case, from the fitting procedure with the function $y=O+Ax^{2}+$B$x^{3}$, we extract a ratio A/B = 1.25±0.37, in agreement with the experimental ratio 1.296 (see Table 1 of the main text) between the SHG and THG CEs that we have found experimentally.

**Figure S10:** Signal measured on sample A (a) and H1(b) by the Si-bolometer with the experimental configuration of fig.3a of the main article, while keeping the FTIR moving mirror at a fixed position, as a function of QCL pump power, after filtering the QCL fundamental line with a 1mm thick Ta-filter (black dots). Fit procedure on the experimental data, perfomed with a 3^rd^ order polynomial curve in **(a)**: $y=o+ax^{3}$, with o = -0.48 ± 0.07, a = 0. 098±0.007; in **(b)**: $y=O+Ax^{2}+Bx^{3}$, with O = -0.87 ± 0.6, A= 0.10±0.36 and B = 0.079±0.011 (red lines).

**S12. Atomic force microscope on the VdW heterostructure**

**The atomic force microscope images measured on sample H1 and H2 (Fig. S11) reveals mean roughness of 1.75 nm on sample H1 and 2.16 nm on sample H2.

**Figure S11**: Topography of the InBiSe/BiSe heterostructure TI films, sample H1 (top) and H2 (bottom). From the image analysis, we extract a mean roughness of 1.75nm on sample H1 and 2.16 nm on sample H2.

**References**

1. Pistore, V. *et al.* Terahertz Plasmon Polaritons in Large Area Bi2Se3 Topological Insulators. *Adv. Opt. Mater.* **n/a**, 2301673 (2023).

2. Pogna, E. A. A. *et al.* Mapping propagation of collective modes in Bi2Se3 and Bi2Te2.2Se0.8 topological insulators by near-field terahertz nanoscopy. *Nat. Commun.* **12**, 6672 (2021).

3. Di Pietro, P. *et al.* Terahertz Tuning of Dirac Plasmons in Bi2Se3 Topological Insulator. *Phys. Rev. Lett.* **124**, (2020).

4. Tielrooij, K.-J. *et al.* Milliwatt terahertz harmonic generation from topological insulator metamaterials. *Light Sci. Appl.* **11**, 315 (2022).

5. Hafez, H. A. *et al.* Terahertz Nonlinear Optics of Graphene: From Saturable Absorption to High-Harmonics Generation. *Adv. Opt. Mater.* **8**, 1900771 (2020).

6. Han, J. W. *et al.* Plasmonic Terahertz Nonlinearity in Graphene Disks. *Adv. Photonics Res.* **3**, 2100218 (2022).

7. Mics, Z. *et al.* Thermodynamic picture of ultrafast charge transport in graphene. *Nat. Commun.* **6**, 7655 (2015).

8. Tomadin, A. *et al.* The ultrafast dynamics and conductivity of photoexcited graphene at different Fermi energies. *Sci. Adv.* **4**, eaar5313 (2023).

9. Massicotte, M., Soavi, G., Principi, A. & Tielrooij, K.-J. Hot carriers in graphene – fundamentals and applications. *Nanoscale* **13**, 8376–8411 (2021).

10. Di Gaspare, A. *et al.* Electrically Tunable Nonlinearity at 3.2 Terahertz in Single-Layer Graphene. *ACS Photonics* (2023) doi:10.1021/acsphotonics.3c00543.

11. Sobota, J. A. *et al.* Ultrafast electron dynamics in the topological insulator Bi_2_Se_3_ studied by time-resolved photoemission spectroscopy. *J. Electron Spectros. Relat. Phenomena* **195**, 249–257 (2014).

12. Cox, J. D., Marini, A. & de Abajo, F. J. G. Plasmon-assisted high-harmonic generation in graphene. *Nat. Commun.* **8**, 14380 (2017).

13. Jadidi, M. M. *et al.* Tunable Terahertz Hybrid Metal–Graphene Plasmons. *Nano Lett.* **15**, 7099–7104 (2015).

14. Wang, Y. & Law, S. Optical properties of (Bi_1-x_In_x_)_2_Se_3_ thin films. *Opt. Mater. Express* **8**, 2570–2578 (2018).

15. Chudpooti, N. *et al.* Wideband dielectric properties of silicon and glass substrates for terahertz integrated circuits and microsystems. *Mater. Res. Express* **8**, (2021).

16. Richter, W., Kohler, H. & Becker, C. A Raman and Far-Infrared Investigation of Phonons. *Phys. Stat. sol* **84**, 619 (1977).

17. Spirito, D. *et al.* Weak antilocalization and spin-orbit interaction in a two-dimensional electron gas. *Phys. Rev. B* **85**, 235314 (2012).

18. Zhang, Y., Tan, Y.-W., Stormer, H. L. & Kim, P. Experimental observation of the quantum Hall effect and Berry’s phase in graphene. *Nature* **438**, 201–204 (2005).
